# Beyond Free Virions: Interconnected Secretory Pathways and Reticulon 3 (RTN3) Coordinate Extracellular Vesicle Diversity for Infectious Exosome Generation

**DOI:** 10.3390/biology15090701

**Published:** 2026-04-29

**Authors:** Razieh Bitazar, Clinton Njinju Asaba, Arnaldo Nakamura, Tatiana Noumi, Patrick Labonté, Terence Ndonyi Bukong

**Affiliations:** Armand-Frappier Santé Biotechnologie Research Center, Institut National de la Recherche Scientifique, Laval, QC H7V 1B7, Canada; razieh.bitazar@inrs.ca (R.B.); clinton.asaba@inrs.ca (C.N.A.);

**Keywords:** extracellular vesicles, infectious exosomes, reticulon 3 (RTN3), dengue virus, multivesicular bodies, secretory autophagy, endoplasmic reticulum contact sites, host–pathogen interactions

## Abstract

Extracellular vesicles (EVs) can disseminate replication-competent viral genomes complexed with selected host proteins, enabling stealth cell-to-cell transfer within lipid membrane-enclosed bubbles. In addition to complementing free-virion spread, EV-associated genomes can be protected from neutralizing antibodies and persist under conditions in which classical virion production decreases. Here, we propose a route-resolved framework in which interconnected cellular secretory pathways, including endoplasmic reticulum (ER) remodeling, multivesicular body (MVB) biogenesis, secretory autophagy, and plasma-membrane budding, jointly generate EV heterogeneity and create discrete opportunities for the capture, protection, and export of infectious cargo. We highlight reticulon-3 (RTN3), an ER-shaping protein, as an upstream regulator that can couple infection-induced ER microdomains to endosomal docking and to autophagy-linked trafficking decisions that bias intermediates toward secretion rather than degradation. Supporting this view, transmission electron microscopy of dengue virus-infected cells reveals extensive vesicular remodeling, including irregular MVBs adjacent to the plasma membrane and autophagosome-like double-membrane structures, consistent with altered vesicular routing following RTN3 perturbation. Collectively, these route-resolved, spatially organized spatio-organelle changes support a pathomechanistic model in which RTN3-mediated ER remodeling reshapes ER-endosome-autophagy trafficking interfaces, creating regulated decision points that can be leveraged to stratify infectious EV subsets (with infectivity-linked single-vesicle and quantitative proteomics approaches) and to inform host-directed strategies that curb non-lytic viral dissemination.

## Simple Summary

Viruses do not always spread only as complete virus particles. Some can also move from one cell to another inside tiny membrane-covered packages released by infected cells, which may help them escape parts of the body’s defenses. In this study, we examined how the dengue virus may use several connected transport systems inside the cell to produce these packages. We asked whether a cell protein called reticulon 3 (RTN3) helps control this process. We found that infection caused major changes in the cell’s internal membrane network, including structures involved in packaging, transport, and release. When RTN3 was removed, these structures became abnormal, and the cells showed reduced signs of dengue virus activity. These findings suggest that RTN3 helps infected cells reshape their internal membranes in ways that support the protected release of infectious viral material. Our work is valuable to society because it identifies a possible new treatment strategy. Specifically, instead of targeting only the virus, future therapies may also block the cellular pathways that viruses exploit to spread.

## 1. Introduction

**Figure 1:**
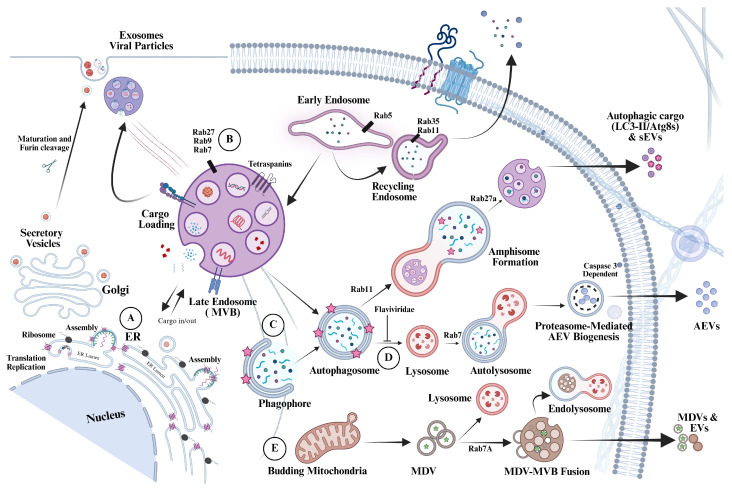
Multifaceted routes of extracellular vesicle (EV) biogenesis and unconventional viral non-lytic egress. (A) Schematic representation of dengue virus translation and replication at the endoplasmic reticulum (ER). Following entry, viral RNA is translated at ER-associated ribosomes, leading to the formation of replication complexes within ER membranes. The violet hairpin elements represent RTN3S, an ER-shaping protein that inserts into the membrane and induces curvature through its wedge-like topology, thereby promoting membrane bending and facilitating the formation of replication-associated vesicular structures. (B) Multivesicular bodies (MVBs) are spatially organized within the central cytoplasm and near multiple organelles, supporting efficient cargo loading and trafficking through Rab5-, Rab7-, Rab9-, and Rab27-associated routes. (C) Autophagosomes can fuse with MVBs to generate amphisomes, hybrid organelles often regulated by Rab11 and Rab27A, that sort autophagic and endosomal cargo toward lysosomal degradation or secretion (pink star), defining a key intersection between autophagy and endosomal EV biogenesis. (D) Apoptotic extracellular vesicles (AEVs) can be produced through a proteasome-dependent pathway within autolysosomal compartments and released from early apoptotic cells in a caspase-3-dependent manner; notably, Flaviviridae has been reported to interfere with phagosome–lysosome fusion, with potential consequences for this degradative-to-secretory balance. (E) Mitochondrial-derived vesicles (MDVs) bud from mitochondria as single- or double-membrane carriers containing associated cargo (green star) and can be routed to MVBs via Rab7-dependent trafficking or delivered to lysosomes for degradation, thereby linking mitochondrial stress responses to EV cargo diversification. This route-resolved framework predicts that EV subpopulations emerging from endoplasmic reticulum (ER)–multivesicular body (MVB) and autophagy-linked intermediates will carry distinguishable protein and proteoform signatures. These signatures are expected to include selective enrichment of membrane-shaping and ribonucleic acid (RNA)-binding factors that stabilize replication-competent cargo and support secretion. Functional correlation of infectivity readouts with quantitative proteomics and orthogonal vesicle stratification will be essential for validating these pathway-specific predictions and defining robust molecular markers of infectious EV subsets.

Extracellular vesicles (EVs) have moved from a niche curiosity to a central feature of host–pathogen biology. In viral infections, EVs complement classical release of free virions by transporting replication-competent viral nucleic acids, proteins, and lipids between cells as membrane-cloaked cargo. Because vesicle membranes can shield labile ribonucleic acid (RNA) from extracellular nucleases and alter uptake pathways, vesicle-mediated transfer may contribute to stealth dissemination in tissues with antibodies present or where virions are rapidly cleared. Notably, EVs are not a single entity but a spectrum of particles produced by distinct biogenesis pathways. Among the various cell-released vesicular structures, including apoptotic bodies and microvesicles (also called ectosomes), exosomes arise as intraluminal vesicles (ILVs) within multivesicular bodies (MVBs). They are released upon MVB fusion with the plasma membrane. Microvesicles bud directly from the plasma membrane through local lipid remodeling and membrane scission. During viral infection and stress, additional EV-like particles can emerge from programmed cell death (apoptotic bodies and blebs), autophagy-linked secretion and a more recently identified class of intracellular vesicles derived from mitochondria (MDVs), expanding both EV diversity and the opportunities for viral cargo to exit cells [1,2] (Figure 1). Within the endosomal pathway, ILV formation can proceed through endosomal sorting complexes required for transport (ESCRT) machinery or through ESCRT-independent mechanisms that rely on lipid and tetraspanin organization. Rab family small guanosine triphosphatases (Rab GTPases) coordinate late endosomal maturation and determine whether MVBs are directed to lysosomal degradation or to secretion. Rab7A, Rab11, Rab27A, Rab27B, and Rab35 are among the regulators that promote MVB positioning, docking, and fusion with the plasma membrane, whereas Rab5 and Rab7 also support downstream endocytic trafficking after MVB biogenesis. These controls provide multiple points at which viruses can exploit vesicle fate to generate and release infectious viral exosomes. Several viruses are known to hijack ESCRT components to facilitate their budding and amplify vesicular egress. At the same time, alternative ILV biogenesis routes can also support cargo loading that is not strictly dependent on ubiquitin. Tetraspanin oligomerization, sphingomyelinase-driven ceramide production, phospholipase D2 (PLD2) activity, and ADP-ribosylation factor 6 (ARF6)-dependent remodeling have all been implicated in ILV or microvesicle formation. A practical consequence is that vesicle-mediated dissemination is unlikely to be blocked by inhibiting a single pathway. Instead, the relevant question becomes which steps are selectively required to package infectious viral material inside infectious exosomes. It is most likely that cargo selection and incorporation into EVs is not random. Infectious cargo must first be enriched at sites of vesicle formation and then captured by sorting factors that direct it into release-competent compartments. In addition to these molecular sorting mechanisms, emerging evidence indicates that spatial membrane organization also contributes to cargo selection. In particular, membrane contact sites (MCSs) between the endoplasmic reticulum (ER) and organelles such as late endosomes, autophagosomes, multivesicular endosomes (MVEs), or the plasma membrane have been identified as important regulators of EV cargo loading and sorting. From this perspective, the long isoform of RTN3 has been shown to facilitate membrane contact sites between the endoplasmic reticulum and endosomes, thereby contributing to endosomal maturation and cargo trafficking, suggesting a possible role in EV cargo loading. The idea that direct membrane contact sites between the ER and the plasma membrane may facilitate EV or virus secretion represents an intriguing hypothesis that warrants further investigation. Furthermore, selective loading can be mediated by protein complexes, lipid partitioning, or by adaptor proteins that bind specific RNAs or proteins. Also, several mechanisms that concentrate cytosolic cargo, including recruitment by RNA-binding proteins (RBPs) and by ubiquitin ligases that act as scaffolds for capture, have been advanced. These include fragile X messenger ribonucleoprotein protein 1 (FMR1), the tripartite motif-containing ubiquitin ligase 25 (TRIM25), and lysosome-associated membrane protein 2A (LAMP2A), amongst others, which have been linked to microautophagy or chaperone-mediated routes that can intersect with EV biogenesis. Autophagy provides an additional layer of selectivity and plasticity in EV production (Figure 1). Canonical autophagy generates double-membrane autophagosomes that can fuse with lysosomes for degradation. In contrast, during secretory autophagy, autophagosomes are diverted toward secretion. As such, autophagosomes can merge with endosomal compartments to form amphisomes, which are hybrid organelles marked by microtubule-associated protein 1A/1B-light chain 3 (LC3) and the exosomal marker CD63. Subsequent fusion of amphisomes or autophagosomes with the plasma membrane can release EV-like particles, including LC3-associated vesicles and autophagosome-like microvesicles (ALMVs), thereby exporting cytosolic material while bypassing lysosomal degradation. This non-canonical autophagy-related mechanism expands the repertoire of EV release routes, creating additional opportunities for viruses to disseminate infection via infectious EVs. A possible mechanism is that, early in infection, viral replication generates abundant RNA–protein complexes that must be stabilized and sorted rather than degraded. Autophagy-related genes (ATGs) are then recruited to the endosomal or plasma membranes via LC3-associated phagocytosis and endosomal microautophagy, thereby placing LC3-decorated membrane domains at key sorting sites.

LC3 recruitment at these sites can bias multivesicular bodies (MVBs) toward nonterminal maturation by limiting or delaying MVB acidification, thereby preserving cargo integrity and increasing the probability of secretion. In parallel, LC3 can act as a molecular ‘handle’ for selective sorting/loading by engaging RNA-binding proteins (RBPs) that recognize viral or proviral RNA features, suggesting that RNA selection is coupled to the identity of the LC3-marked membrane [3]. As a result, a tunable decision point between degradation and secretion can be shifted toward secretion, allowing replication-competent viral genomes to be packaged into EVs and released as protected, delivery-ready infectious EVs [4].

A key unresolved knowledge gap is the spatiotemporal location within cells at which infectious cargo is selected and committed to the exosomal release pathway. It remains unclear whether this selection occurs at defined endoplasmic reticulum (ER)-associated membrane contact sites, during early endosome maturation, or later multivesicular body sorting stages. Defining these spatial routes and timing is essential to explain how infectious material is preferentially incorporated into exosomes rather than diverted into degradative trafficking pathways. One possibility is that replication-competent viral ribonucleoprotein complexes are specified at replication sites by RNA-binding proteins (RBPs) together with host sorting adaptors and then delivered to endosomes [5]. A second plausible model is that selection happens later at LC3-decorated MVBs. In this case, LC3-associated membranes would recruit RBPs and coordinate with endosomal trafficking machinery [3]. Such coordination could limit MVB acidification and favor secretion over degradation. It is well established that viral replication in most RNA viruses is tightly linked to ER membrane remodeling, which creates replication compartments and curvature-rich scaffolds. We propose a scenario in which early membrane architecture, rather than being a passive backdrop, can shape downstream vesicle routing. We further provide evidence supporting a key role for RTN3 in coordinating ER membrane remodeling with vesicle biogenesis and trafficking, thereby influencing the formation and functional properties of extracellular vesicles during infection.

## 2. Materials and Methods

### 2.1. Huh7 Cell Culture, Transduction and Infection Experiments

Huh7 hepatoma cells (a gift from Dr. Charlie Rice, Rockefeller University, New York, NY, USA) [6,7] available commercially from Research Bioresources Cell Bank, catalogue number JCRB0403 and Cellosaurus accession number CVCL_0336, were cultured in Dulbecco’s Modified Eagle Medium (DMEM) (Thermo Fisher Scientific, Cleveland, OH, USA) supplemented with 1% penicillin/streptomycin (P/S) (10,000 U/mL penicillin and 10,000 µg/mL streptomycin; Gibco, Waltham, MA, USA, REF No. 15140-122), 10 mM HEPES buffer (Gibco, REF No. 15630-080), and 10% fetal bovine serum (FBS; Gibco, Cat. No. A5256501). For transduction, 2 × 10^5^ cells were seeded in 6-well plates and transduced with lentivirus at a multiplicity of infection (MOI) of 5 in the presence of 8 µg/mL polybrene. After 24 h of transduction at 37 °C with 5% CO_2_, the medium was replaced with fresh complete medium, and the cells were cultured for an additional 24 h. After 48 h of transduction, cells were infected with dengue virus type 2 (New Guinea C strain, ATCC VR-1584TM) at a multiplicity of infection (MOI) of 1. Cells were harvested at 72 h post-infection for subsequent downstream analyses. All experiments with pathogenic materials were conducted in our Biosafety Level 2 (BSL2) laboratory located at the Institut National de la Recherche Scientifique—Centre Armand-Frappier Santé Biotechnologie (INRS-CAFSB), Canada.

### 2.2. CRISPR/Cas9 Knockout and Lentiviral Transduction

RTN3S-specific gRNAs (5′CTCGGCTCCGAAGGACGACG3′) were designed using the CHOPCHOP web tool (http://chopchop.cbu.uib.no, accessed on 25 April 2026) and cloned into the lentiCRISPRv2 plasmid (Addgene #52961). Lentiviral particles were generated by co-transfecting lentiCRISPRv2 with psPAX2 and pMD2.G plasmids in HEK293T cells (Figure S1). Viral supernatants were collected, filtered through 0.45 μm filters (Millipore Sigma, Burlington, MA, USA), and stored at −80 °C. For titration, HeLa cells were infected with serial dilutions of the virus and selected with puromycin (1 µg/mL). Surviving colonies were stained with crystal violet and counted. Huh7 cells were transduced with lentivirus at an MOI of 5–10 in the presence of 8 μg/mL polybrene to adjust the functional titer.

The original western blot images for Figure S1 are available in File S1.

### 2.3. Quantitative Real-Time PCR Assay and Immunoblotting

Huh7 hepatoma cells (1.5 × 10^4^ cells) were cultured in Dulbecco’s Modified Eagle Medium (DMEM; Thermo Fisher Scientific, Cleveland, OH, USA) supplemented with 1% penicillin/streptomycin (10,000 U/mL penicillin and 10,000 μg/mL streptomycin; Gibco, REF No. 15140-122), 10 mM HEPES buffer (Gibco, REF No. 15630-080), and 10% exosome-depleted fetal bovine serum (FBS; Gibco, Cat. No. A2720801). Cells were transduced with lentivirus at a multiplicity of infection (MOI) of 5 in the presence of 8 μg/mL polybrene for 48 h. Subsequently, cells were infected with the dengue virus at an MOI of 1. Following infection, cells were harvested and subjected to downstream quantitative PCR (qPCR) and immunoblotting analyses. Cell pellets were lysed, and total RNA was recovered using the RNeasy Micro Kit (Qiagen, Cat. No. 74104, Hilden, Germany) according to the supplier’s instructions. One microgram of RNA from each sample was converted to cDNA with the iScript™ Reverse Transcription Supermix (Bio-Rad, Hercules, CA, USA). The resulting cDNA served as a template for SYBR Green-based quantitative PCR (qPCR) on a Bio-Rad CFX96 instrument. Concomitantly, cells were lysed on ice using RIPA buffer containing protease inhibitors (cOmplete™, Mini; Roche Applied Science, Indianapolis, IN, USA, Cat. #: 11836153001). The RIPA buffer (Boston Bioproducts, Milford, MA, USA, Cat. #: BP-115D). Lysates were incubated on ice for 30 min with occasional gentle mixing, briefly vortexed, and then kept on ice for another 5 min before being centrifuged at 14,000 rpm (Eppendorf 5417R centrifuge, Hamburg, Germany; ~17,750× *g*) for 20 min at 4 °C. The resulting supernatants were collected, total protein levels measured using a BCA assay, and aliquots stored for later immunoblotting. RTN3S was identified using ProteinTech (Rosemont, IL, USA, Cat. #: 12055-2-AP), and DENV NS3 was detected with GeneTexantibody (Irvine, CA, USA, Cat. #: GTX124252).

### 2.4. Transmission Electron Microscopy (TEM)

Cell pellets were post-fixed in freshly prepared 1.3% (*w*/*v*) osmium tetroxide in collidine buffer containing potassium ferrocyanide for 1 h at 4 °C, followed by three washes (5 min each) in distilled water. Samples were then post-fixed and counterstained with 1% aqueous uranyl acetate for 30 min at room temperature in the dark, followed by three additional washes in distilled water. Specimens were dehydrated through a graded acetone series (25%, 50%, 75%, and 95% acetone in water; 30 min each), followed by two changes of 100% acetone (≥30 min each). Dehydrated samples were infiltrated in a 1:1 mixture of SPURR resin (EM0300-1KT; Sigma-Aldrich, St. Louis, MO, USA) and acetone for 16–18 h, then embedded through two successive incubations in pure SPURR resin (≥2 h each). Samples were cut into small pieces, transferred into BEEM capsules (size 00, BEEM Inc., Bronx, New York, NY, USA), filled with fresh resin, and allowed to stand at room temperature for 16–18 h before polymerization at 60–65 °C for 20–30 h. Ultrathin sections (60–90 nm) were cut using an ultramicrotome (Leica UC7, Wetzlar, Germany) and mounted on 200-mesh copper grids. Sections were contrasted with uranyl acetate in 50% ethanol (20–25 min) followed by lead citrate staining (5–7 min). Imaging was performed using a transmission electron microscope (Hitachi HT7800, Tokyo, Japan) equipped with an AMT Nanosprint II camera (Advanced Microscopy Techniques, Woburn, MA, USA).

## 3. Results and Discussion

RNA viruses have evolved remarkably flexible strategies to remodel host endomembrane systems, not only to sustain intracellular replication but also to enable non-lytic dissemination through heterogeneous extracellular vesicle pathways. Within this broader paradigm, dengue virus is increasingly viewed as exploiting an interconnected continuum of ER-derived replication niches, endosomal and autophagy-linked intermediates, and other vesiculogenic routes to generate distinct EV populations that may differentially protect, transport, and release replication-competent viral cargo (Figure 1). This conceptual framework positions membrane-sculpting host factors as pivotal organizers of viral egress, cargo selectivity, and vesicle infectivity.

Reticulon-3 (RTN3) is an ER-shaping protein that stabilizes high-curvature tubules and can influence ER contact sites with other organelles [8]. By modulating ER topology at the origin of infection-associated membranes, RTN3 could create privileged platforms for the capture of viral ribonucleoprotein complexes and their handoff into endosomal or autophagy-mediated routes. Western blotting validation first confirmed effective RTN3 knockout in the edited Huh7 cells. Immunoblot analysis showed reduced RTN3S expression together with diminished DENV NS3 signal in RTN3 knockout cells, and RT-qPCR similarly showed reduced RTN3S transcript abundance relative to wild-type infected cells (Figure S2).

**Figure 2:**
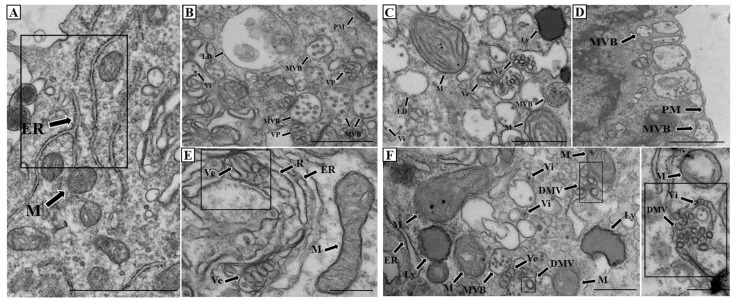
Ultrastructural remodeling of the vesicular network in dengue virus-infected cells. (**A**) Representative transmission electron microscopy (TEM) image of Huh7 control cells. The endoplasmic reticulum (ER) and mitochondria (M) are indicated. The boxed region highlights the close spatial organization of ER membranes and mitochondria within the cytoplasm. Scale bar: 1 μm (**B**) Representative transmission electron microscopy (TEM) image showing a highly vesiculated cytoplasm containing endoplasmic reticulum (ER) membranes, multivesicular bodies (MVBs) close to the plasma membrane (PM), lipid droplets (LD), Vesicle packets (Vp), and virus-induced electron-dense particles (Vi), consistent with virus-induced intracellular membrane remodeling. Scale bar: 500 nm. (**C**) The cytoplasm displays extensive membrane rearrangement, with virus-induced electron-dense particles (Vi) and virus-induced vesicles (Ve) tightly associated with lysosomal structures (Ly), a giant mitochondrion (M), and MVB-enclosed infection-associated cargo, suggesting active vesicular trafficking and organelle remodeling. Scale bar: 500 nm. (**D**) TEM image showing altered MVB morphology positioned adjacent to the plasma membrane (PM) in reticulon-3 (RTN3) knockout cells, consistent with modified vesicle docking and membrane dynamics. Scale bar: 250 nm (**E**) TEM image showing RTN3 knockout cells, elongated virus-induced replication vesicles and ribosome (R)-studded membranes visible in the surrounding rough ER. Scale bar: 250 nm. (**F**) Examples of double-membrane vesicles (DMV) found in dengue-infected cells adjacent to the mitochondria (M). Scale bar: 250 nm. Quantification was based on two independent experiments, with five cells analyzed per condition in each experiment (total n = 10 cells per condition).

Ultrastructural phenotypes in dengue virus-infected cells support this RTN3-centered view. Transmission electron microscopy (TEM) revealed a highly vesiculated cytoplasm containing ER membranes, MVBs, lipid droplets and vesicular structures consistent with virus-induced membrane remodeling during dengue virus infection (Figure 2B). Dengue-infected cells also displayed electron-dense viral particles and electron-dense lysosomal structures, suggesting active trafficking and organelle turnover (Figure 2C). Notably, RTN3 knockout cells exhibit MVBs with irregular morphology positioned adjacent to the plasma membrane, consistent with altered docking or fusion dynamics (Figure 2D). While RTN3 knockout impairs viral replication, it also alters membrane morphogenesis, resulting in more elongated replication vesicles per vesicle packet than in infected cells (Figure 2E and Figure S3).

Previous ultrastructural studies of dengue virus infection have established that viral replication occurs within ER-derived vesicle packets, accompanied by convoluted membranes and the accumulation of immature virions in the ER lumen [9]. Notably, in that study, siRNA-mediated depletion of RTN3 impaired the formation and organization of these replication-associated membranes, highlighting its role in shaping ER architecture during infection. Consistent with these reports, RTN3-competent infected cells in our study displayed canonical DENV-induced membrane remodeling, confirming that our system recapitulates known features of flaviviral infection. In contrast, RTN3 knockout led to distinct ultrastructural alterations not typically observed in standard DENV infection, including elongated vesicles and abnormal vesicle morphology, as well as an apparent accumulation of immature viral particles (Figure S4). These findings indicate that while DENV infection drives vesicle formation, RTN3 is required to maintain proper membrane architecture and ensure efficient viral maturation, and is essential for the initial formation of replication structures.

Dengue virus infection provides a clear example of how autophagy and lipid metabolism can be coupled to the dissemination of infectious agents via extracellular vesicles (EVs) [1]. Suggestively, dengue infection induces autophagosome formation, which can support viral replication by organizing replication-associated membranes [10]. In parallel, dengue can trigger lipophagy, in which lipid droplets and triglycerides are delivered to autophagic compartments and hydrolyzed into free fatty acids. These fatty acids then fuel beta-oxidation, generating adenosine triphosphate (ATP) to meet the energetic demands of replication [11,12]. As autophagosomes mature, they can intersect with multivesicular bodies (MVBs) to form amphisomes. At this stage, cargo reaches a regulated decision point. Amphisomes can proceed to lysosomal degradation or be rerouted to the plasma membrane for secretion [4]. This balance can be tuned by endosomal trafficking and MVB biogenesis, thereby linking autophagy to the release of selected proteins and nucleic acids in EVs. Recent reports also describe an ER-driven route in which autophagosome-like membranes interface with early endosomes in a Rab22A-dependent manner to generate a distinct intermediate compartment termed ‘rafeesomes’. Secretion of rafeesome-derived intraluminal vesicles (ILVs) expands current EV biogenesis models by placing ER-derived membranes upstream of an endosome-linked release pathway [13,14]. In infection, this route could provide a means to export cargo produced at ER-associated replication sites, bypassing the canonical late endosomal sorting steps. However, these intracellular organelle crosstalks extend beyond just the interface between the ER and endosomes. Mitochondrial-derived vesicles (MDVs) can also be induced under metabolic stress and traffic toward endosomal compartments and MVBs, carrying mitochondrial proteins, lipids, and nucleic acids [15] (Figure 2F). Their convergence with endosomal routes may broaden EV cargo diversity and modulate innate immune signaling, particularly during antiviral responses. In viral infections, this integration could couple cellular stress sensing to EV output, with consequences for cellular communication, immune responses, inflammation, and viral dissemination [16]. From a physiological and pathomechanistic perspective, these intersecting release routes create both therapeutic opportunities and tangible risks. Broad inhibition of extracellular vesicle (EV) biogenesis or secretion could unintentionally impair essential intercellular signaling and compromise tissue homeostasis. At the same time, progress is constrained by practical limitations, including the lack of definitive markers that distinguish infectious EV subpopulations from the broader EV pool, which complicates both diagnostics and targeted drug development. Emerging approaches, including single-vesicle profiling and high-resolution molecular characterization, offer a way to resolve this heterogeneity [17,18].

These methods can move the field beyond bulk EV counts by enabling assays that estimate the proportion of EVs carrying replication-competent cargo, rather than measuring total EV abundance alone. In parallel, progress will depend on route-resolved readouts combined with targeted perturbations of host regulatory targets, thereby enabling specific steps in EV release to be linked to defined outcomes [19]. This distinction is important because lowering overall EV output is not equivalent to reducing the subset of EVs that carry replication-competent viral material, and these two outcomes will most definitely have different mechanistic and translational implications. Our recent reports have implicated reticulon 3 (RTN3) as a host factor that remodels endoplasmic reticulum membranes, potentially favoring the production of infectious EVs. The next priority is to use RTN3 as a mechanistic entry point to test how altered membrane architecture influences where cargo is captured, how it is protected from degradation, and when it is diverted into secretory routes. This logic naturally extends to other host-controlled steps that govern compartment fate, including regulators of multivesicular body maturation and docking and the branch points that steer autophagy-linked intermediates toward lysosomal degradation versus secretion. When these steps are perturbed one at a time and evaluated using route-resolved readouts, it should be possible to separate effects on baseline EV biology from those that specifically amplify vesicle-mediated spread.

Several deep questions will evidently follow, and clarity at each step will refine the direction for advancing the field. Specifically, at what stage is infectious cargo first committed to secretion, and is that commitment reversible as trafficking conditions change? Which aspects of membrane identity and compartmental context determine whether cargo is degraded or exported? How do viruses modulate and fine-tune the degradation-versus-secretion balance without disrupting essential homeostatic EV communication? Additionally, can interventions be designed to act at decision points that bias fate toward inhibiting a specific secretion, rather than broadly suppressing overall EV release? Addressing these questions will require time-resolved experiments that link cellular membrane remodeling and trafficking control to cargo integrity and delivery, while keeping physiological EV functions in mind [19].

Taken together, current evidence argues for moving beyond virion-only models to a view in which infection also spreads via host secretory trafficking routes hijacked to export the viral genome. In this framework, ER organization, RTN3-dependent membrane remodeling, and subsequent vesicle routing are mechanistically linked, helping explain why distinct extracellular vesicle (EV) populations can share similar cargo yet differ in their ability to transmit infection. The central challenge now is to pinpoint when replication-competent cargo is committed to secretion, and which trafficking steps are truly required for release. Resolving these decision points will guide the development of antivirals that complement direct-acting strategies by limiting both classical virion spread and EV-mediated, antibody-shielded dissemination.

## 4. Conclusions

Taken together, this brief report supports a route-resolved model in which RTN3-linked endoplasmic reticulum (ER) remodeling intersects with endosomal and autophagy-associated trafficking pathways to shape the production of infectious extracellular vesicles during dengue virus infection. Orthogonal validation of RTN3 knockout, together with TEM-based morphometry, indicates that RTN3 perturbation is associated with altered MVB architecture and elongation of replication vesicles beyond the baseline ultrastructural changes induced by infection alone. These observations refine the conceptual framework for how membrane remodeling may bias cargo toward secretion rather than degradation and identify RTN3-centered trafficking interfaces as candidate targets for host-directed strategies aimed at limiting non-lytic viral dissemination.

## Data Availability

All data supporting the findings of this study are contained within the article.

## Supplementary Materials

The following supporting information can be downloaded at: https://www.mdpi.com/article/10.3390/biology15090701/s1, Figure S1: Schematic representation of lentiviral constructs used for CRISPR-Cas9-mediated gene editing; Figure S2: RTN3 knockout reduces dengue virus infection and RTN3S expression; Figure S3: Quantification of the multivesicular body (MVB) size and replication vesicle size; Figure S4: Comparison of dengue virion morphology in WT-infected vs RTN3-knockout cells; File S1: Original Blots and Representative TEM Micrograph.
