# Supplementary material for: Beyond Free Virions: Interconnected Secretory Pathways and Reticulon 3 (RTN3) Coordinate Extracellular Vesicle Diversity for Infectious Exosome Generation
Source: Biology (Basel). 2026 Apr 29;15(9):701. doi: 10.3390/biology15090701 (PMC13162583; doi:10.3390/biology15090701)
Supplement: Supplementary file 1 [file biology-15-00701-s001.zip › biology-4219667-supplementary figures.pdf]

Supplementary Files

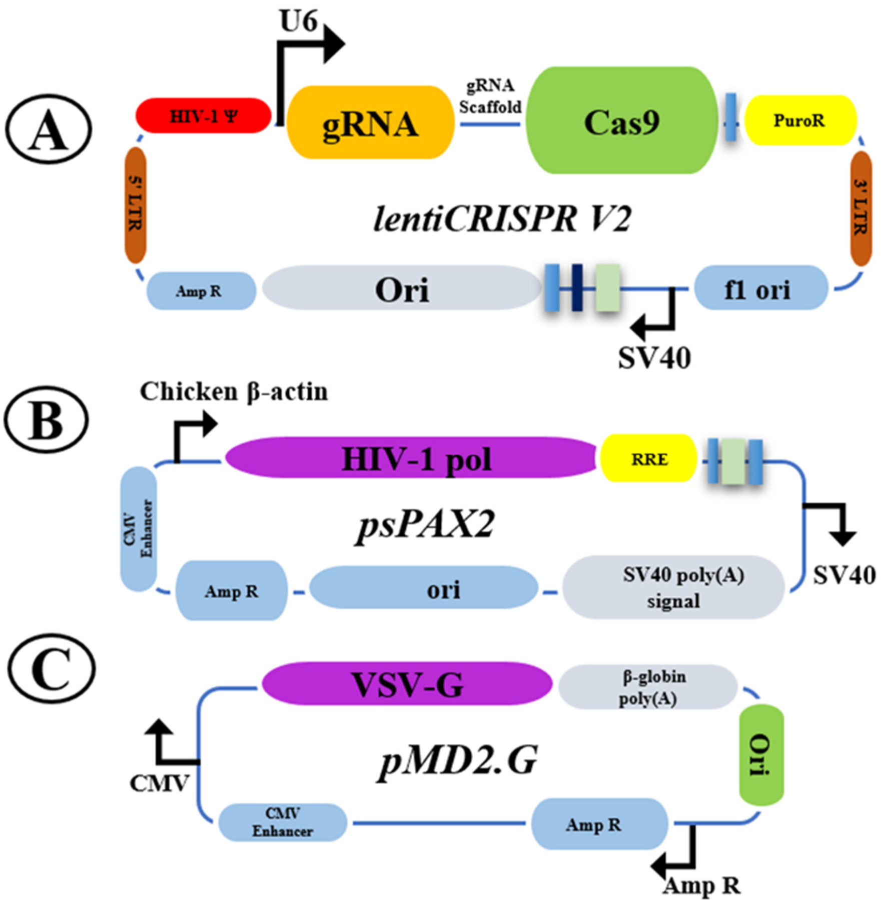

Figure S1. Schematic representation of lentiviral constructs used for CRISPR-Cas9-mediated gene editing. (1) The lentiviral transfer vector **lentiCRISPR v2** expressing the guide RNA (gRNA) under the U6 promoter and Cas9 nuclease, along with a puromycin resistance cassette (PuroR) for selection. (2) The packaging plasmid **psPAX2**, encoding HIV-1 structural and replication proteins (gag/pol), along with regulatory elements required for viral particle assembly. (3) The envelope plasmid **pMD2.G**, expressing the vesicular stomatitis virus glycoprotein (VSV-G) under the CMV promoter to enable pseudotyping of lentiviral particles. These three plasmids are co-transfected to produce lentiviral particles for efficient gene delivery.

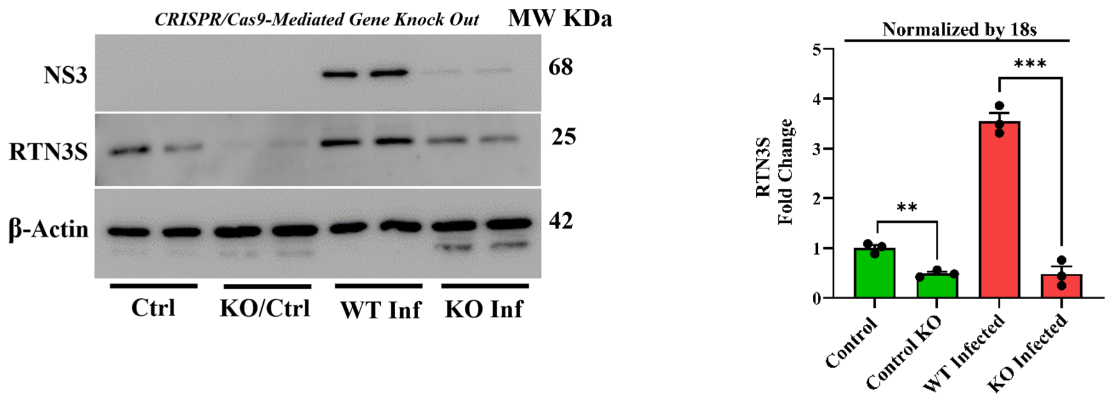

Figure S2. RTN3 knockout reduces dengue virus infection and RTN3S expression.

Left panel: Representative immunoblot analysis showing NS3, RTN3S, and β-actin protein levels in control and RTN3 knockout (KO) cells under uninfected and dengue virus-infected conditions. NS3 serves as a marker of viral infection, while β-actin is used as a loading control. Molecular weight (MW) markers are indicated. **Right panel:** Quantification of RTN3S expression normalized to 18S rRNA, presented as fold change relative to control cells. RTN3S expression is significantly increased in wild-type (WT) infected cells compared to control, whereas RTN3 knockout cells show reduced expression upon infection. Data in right panel represent mean ± SEM of ≥3 independent experiments; statistical significance was determined by Student's t-test (** $p < 0.01$, *** $p < 0.001$).

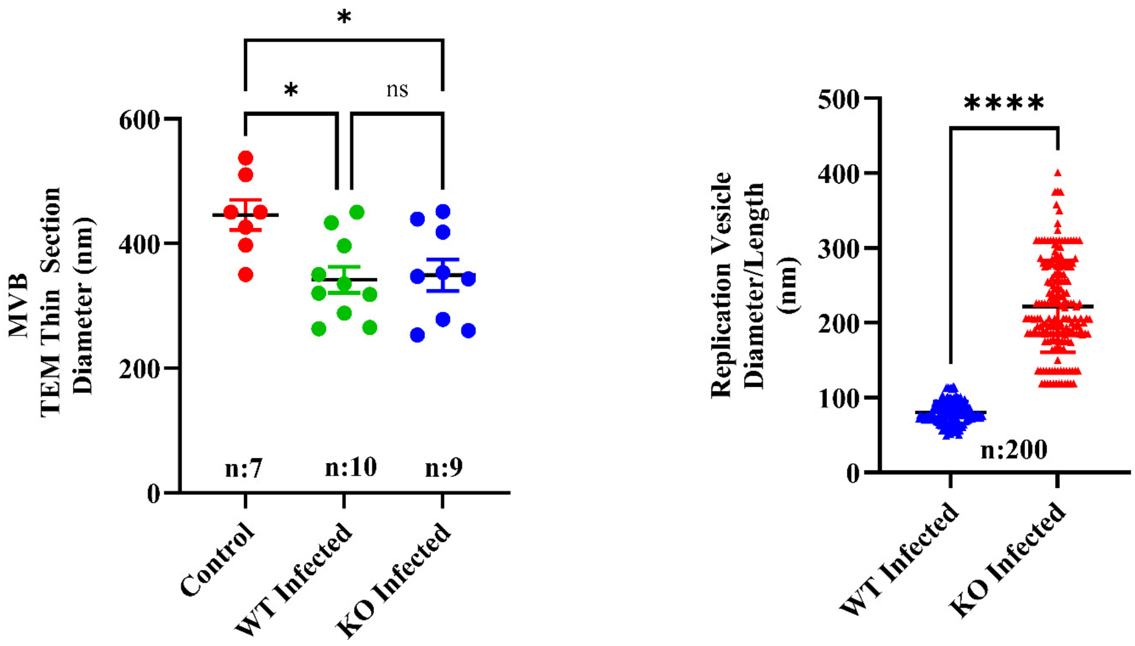

Figure S3. Quantification of the multivesicular body (MVB) size and replication vesicle size.

Left: MVB diameter was measured from TEM images and reported as apparent cross-sectional diameter (nm). Each dot represents one MVB. Data are shown as mean ± SD. Statistical significance was determined using unpaired two-tailed Student's t-test. n = 7 (Control), n = 10 (WT infected), n = 9 (RTN3 KO infected**). Right:** Vesicle size was measured from TEM images and reported as vesicle length/diameter (nm). Each dot represents one vesicle. Data are shown as mean ± SD. Statistical analysis was performed using Mann–Whitney, * $p < 0.05$, ****$p < 0.0001$, ns = not significant. n = 200 vesicles.

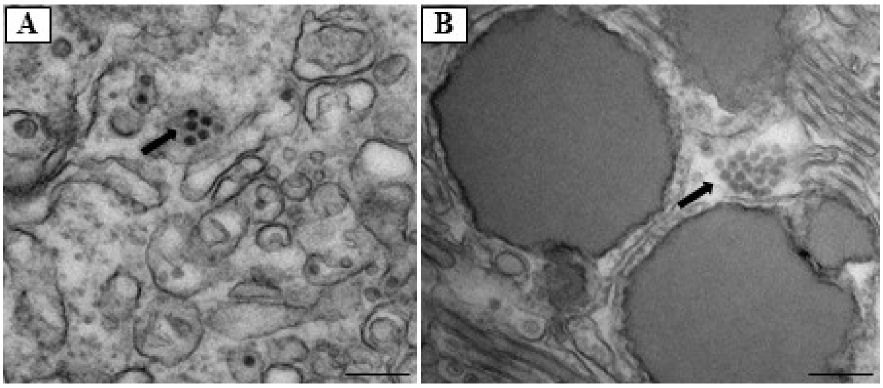

Figure S4. Comparison of dengue virion morphology in WT-infected vs RTN3-knockout cells.

(A) Transmission electron microscopy (TEM) image of dengue-infected Huh7 cells showing clusters of virions within the endoplasmic reticulum (ER), displaying electron-dense cores and relatively smooth outer surfaces. **(B)** RTN3 knockout cells showing accumulation of immature dengue virions (black arrow) within ER-associated compartments. These particles exhibit a fuzzy outer coat and less-defined structure, indicative of impaired maturation and retention within the ER. Scale bars: 250 nm. Viral particles ranged from approximately 50 to 60 nm in diameter.
