# Supplementary material for: Beyond Free Virions: Interconnected Secretory Pathways and Reticulon 3 (RTN3) Coordinate Extracellular Vesicle Diversity for Infectious Exosome Generation
Source: Biology (Basel). 2026 Apr 29;15(9):701. doi: 10.3390/biology15090701 (PMC13162583; doi:10.3390/biology15090701)
Supplement: Supplementary file 1 [file biology-15-00701-s001.zip › biology-4219667-Supplementary File S1_Original Blots and Representative TEM Micrograph.pdf]

Original Blots and Representative TEM Micrograph

Title: Beyond Free Virions: Interconnected Secretory Pathways and Reticulon 3 (RTN3) Coordinate Extracellular Vesicle Diversity for Infectious Exosome Generation

Representative TEM image of whole cell
Huh7 cells infected by dengue virus

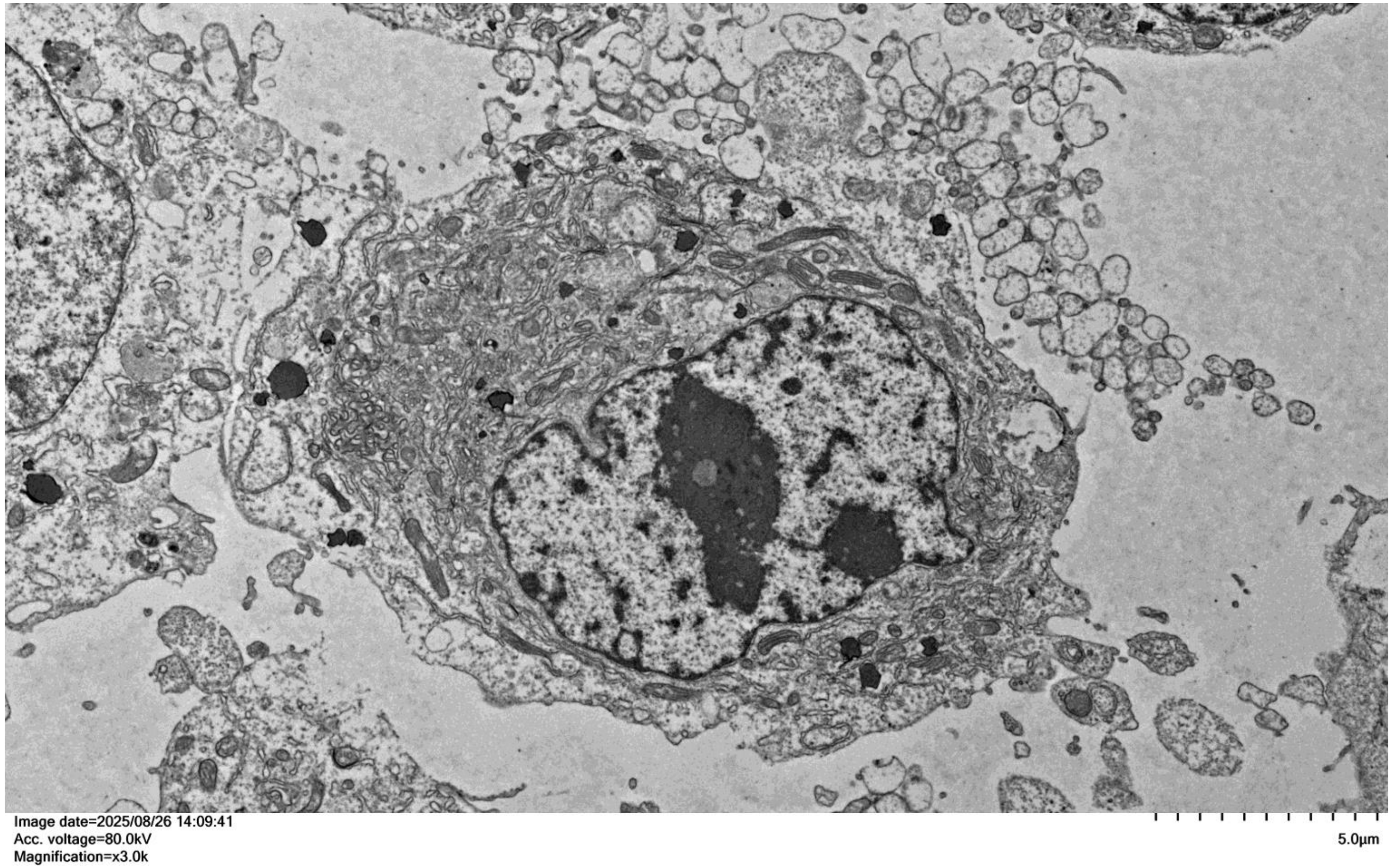

Figure S4.A : Huh7 cells infected by dengue virus

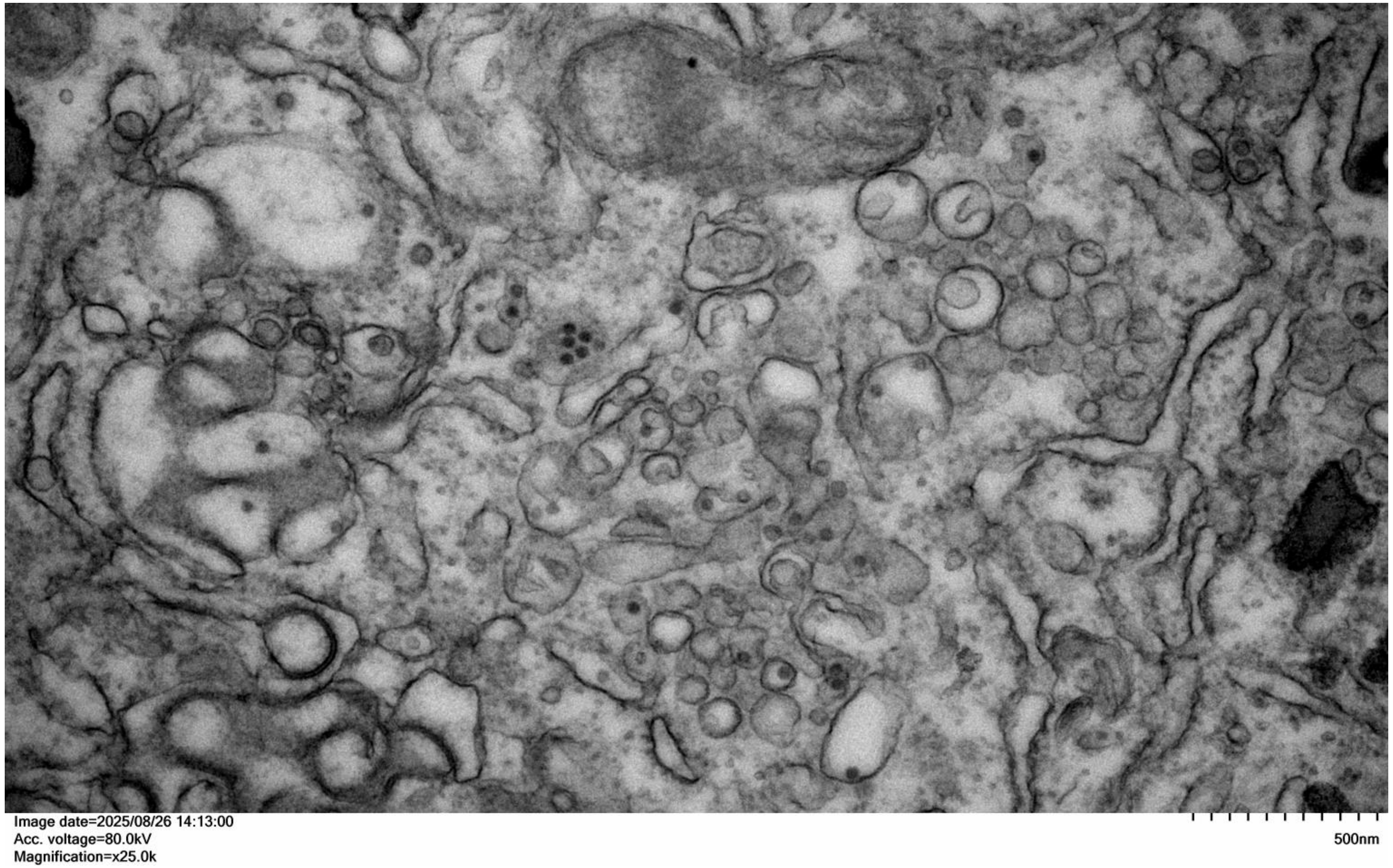

Representative TEM image of whole cell
Huh7 cells infected by dengue virus

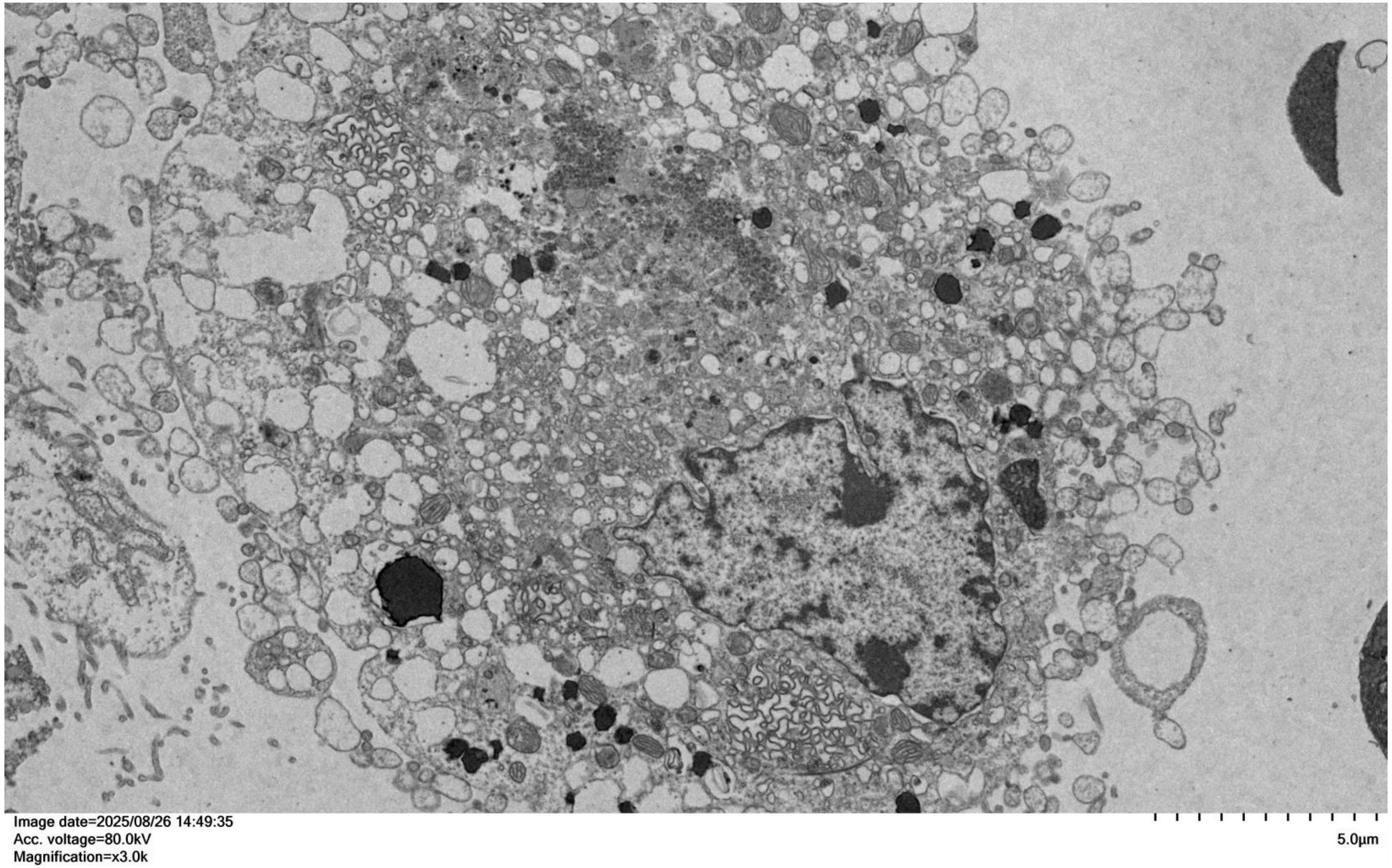

Figure 2C: Huh7 cells infected by dengue virus

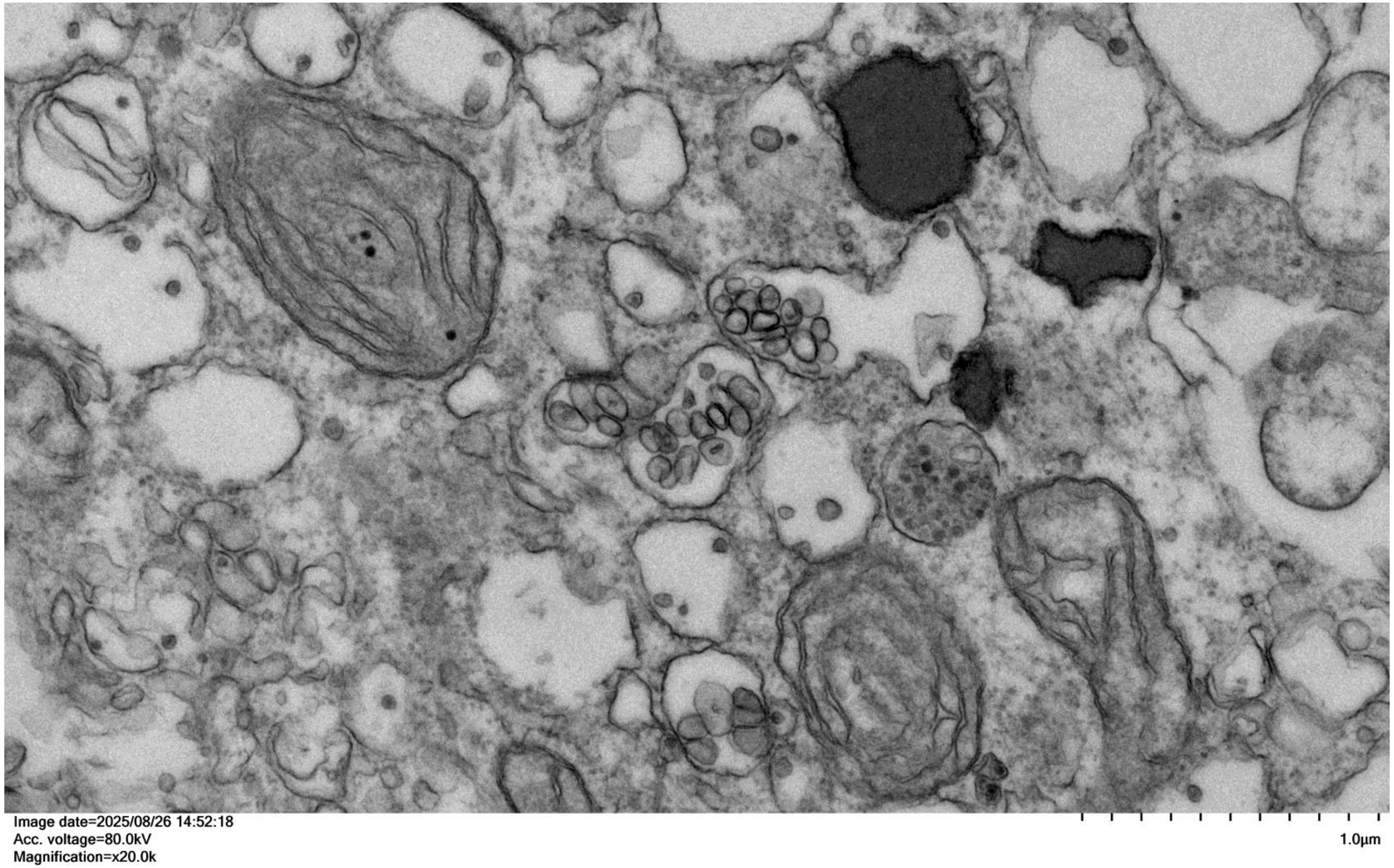

Representative TEM image of whole cell
RTN3 KOHuh7 cells infected by dengue virus

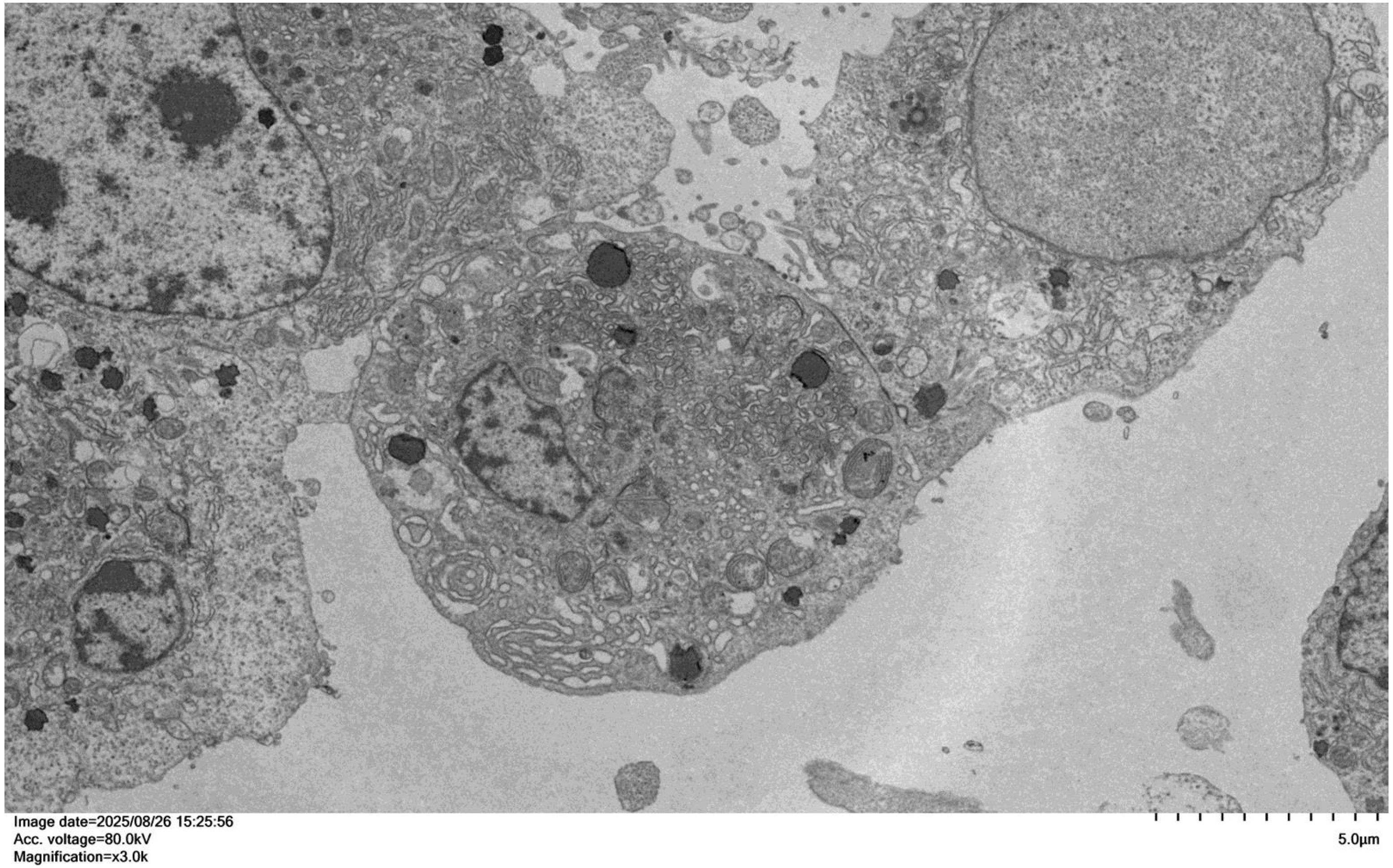

Figure 2E: RTN3 KO/ Huh7 cells infected by dengue virus

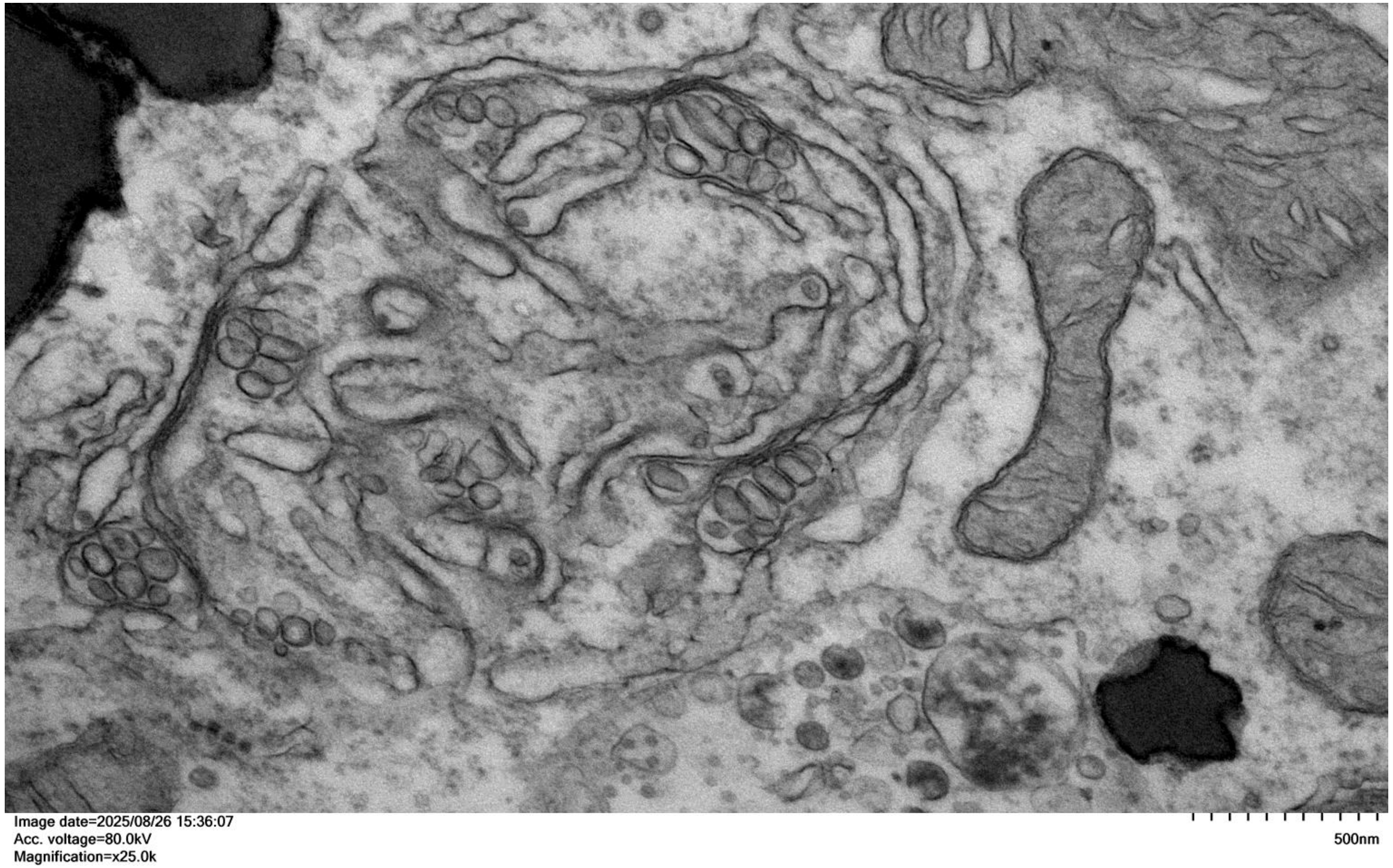

Figure S4.B: RTN3 KO/ Huh7 cells infected by dengue virus

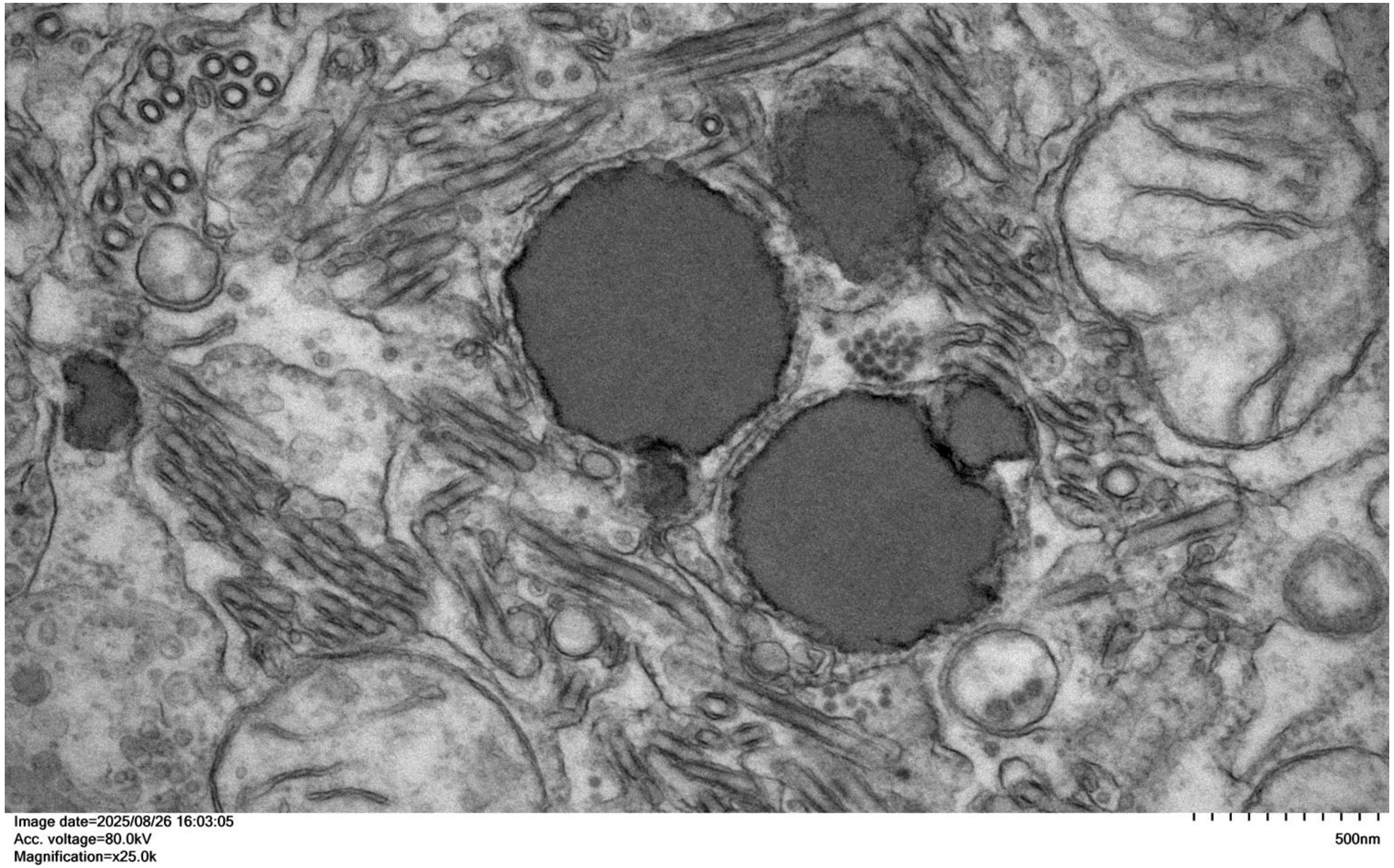

Representative TEM image of whole cell
Huh7 cells infected by dengue virus

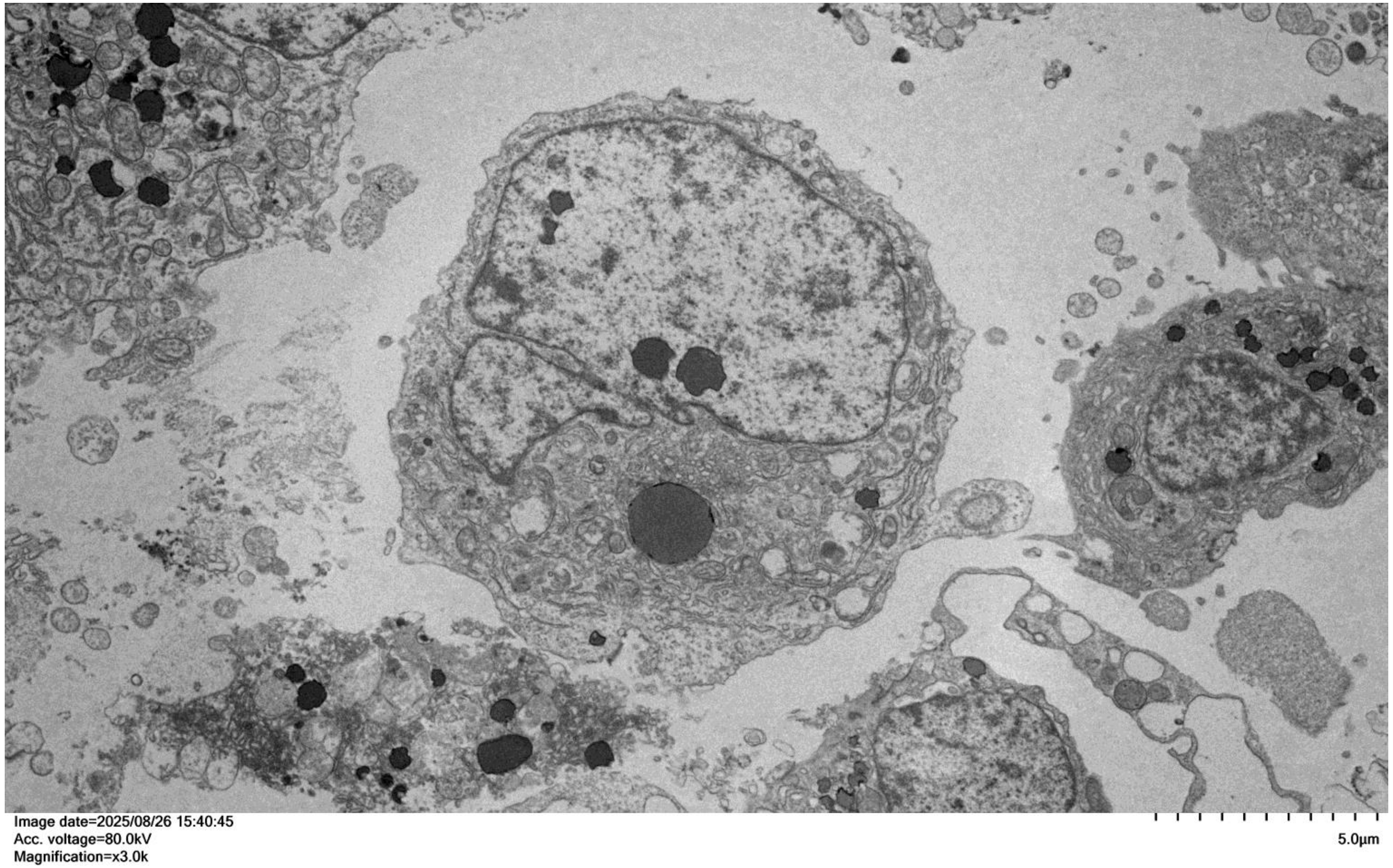

Figure 2B: Huh7 cells infected by dengue virus

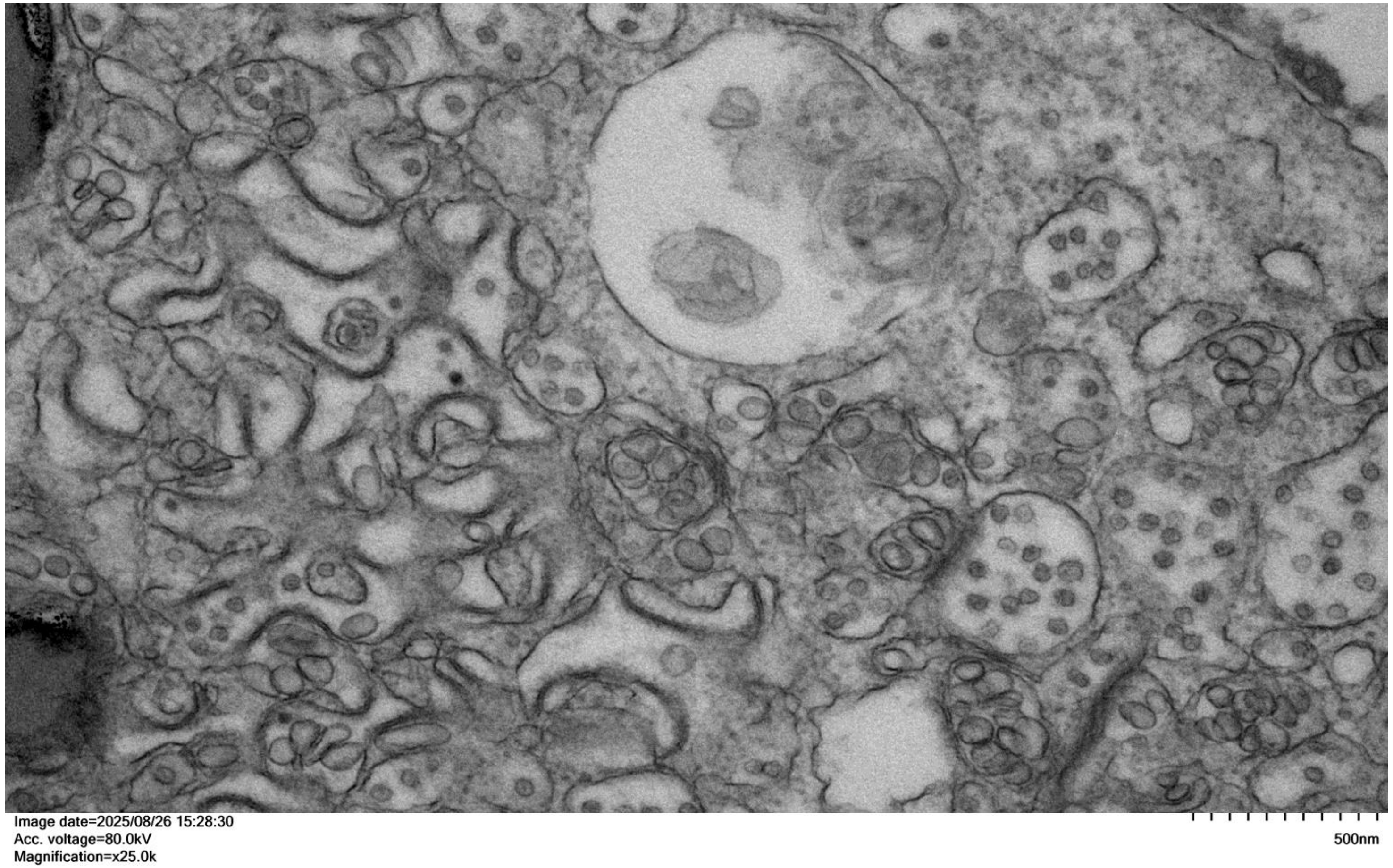

Representative TEM image of whole cell
Huh7 cells infected by dengue virus

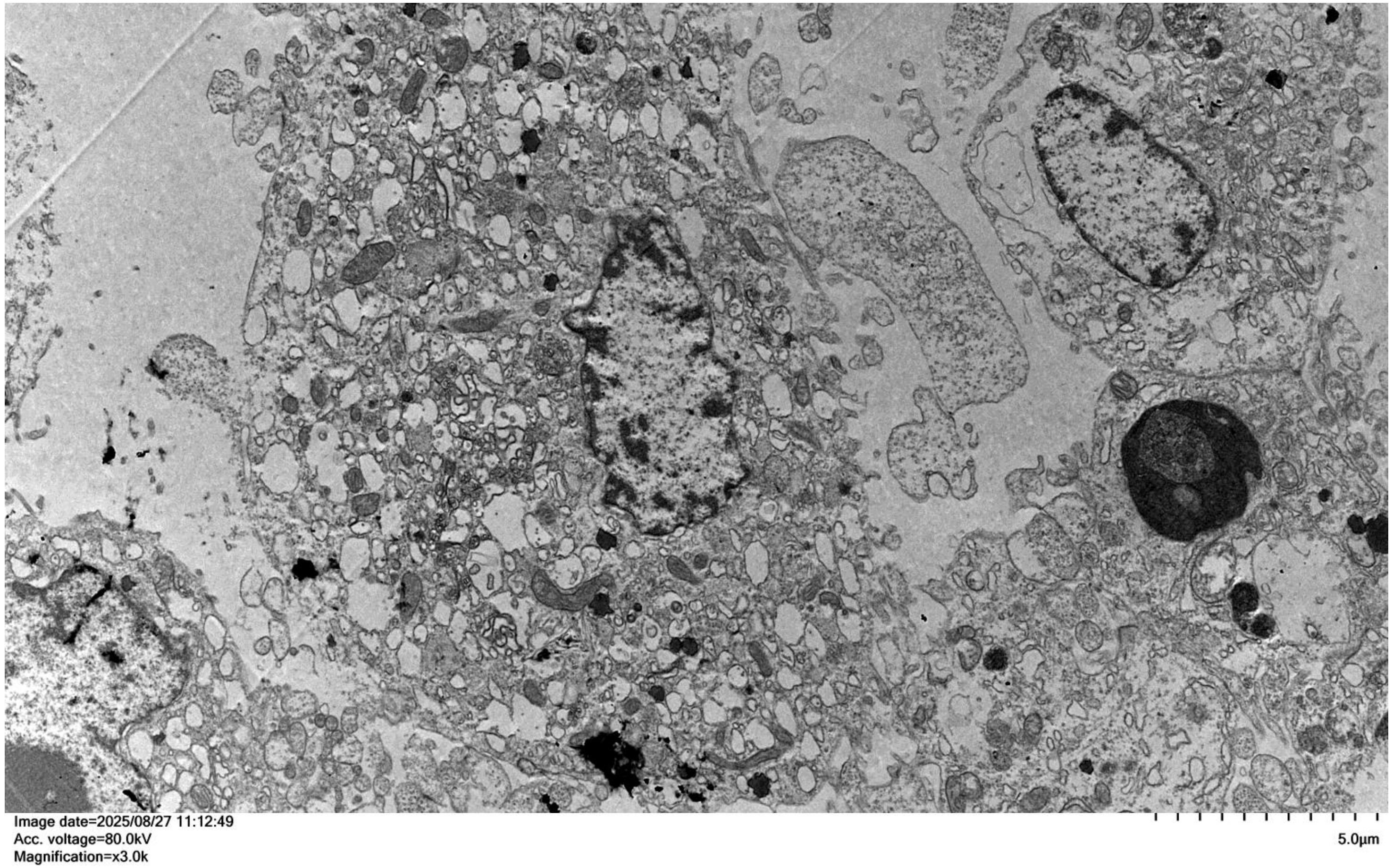

Figure 2F: Huh7 cells infected by dengue virus

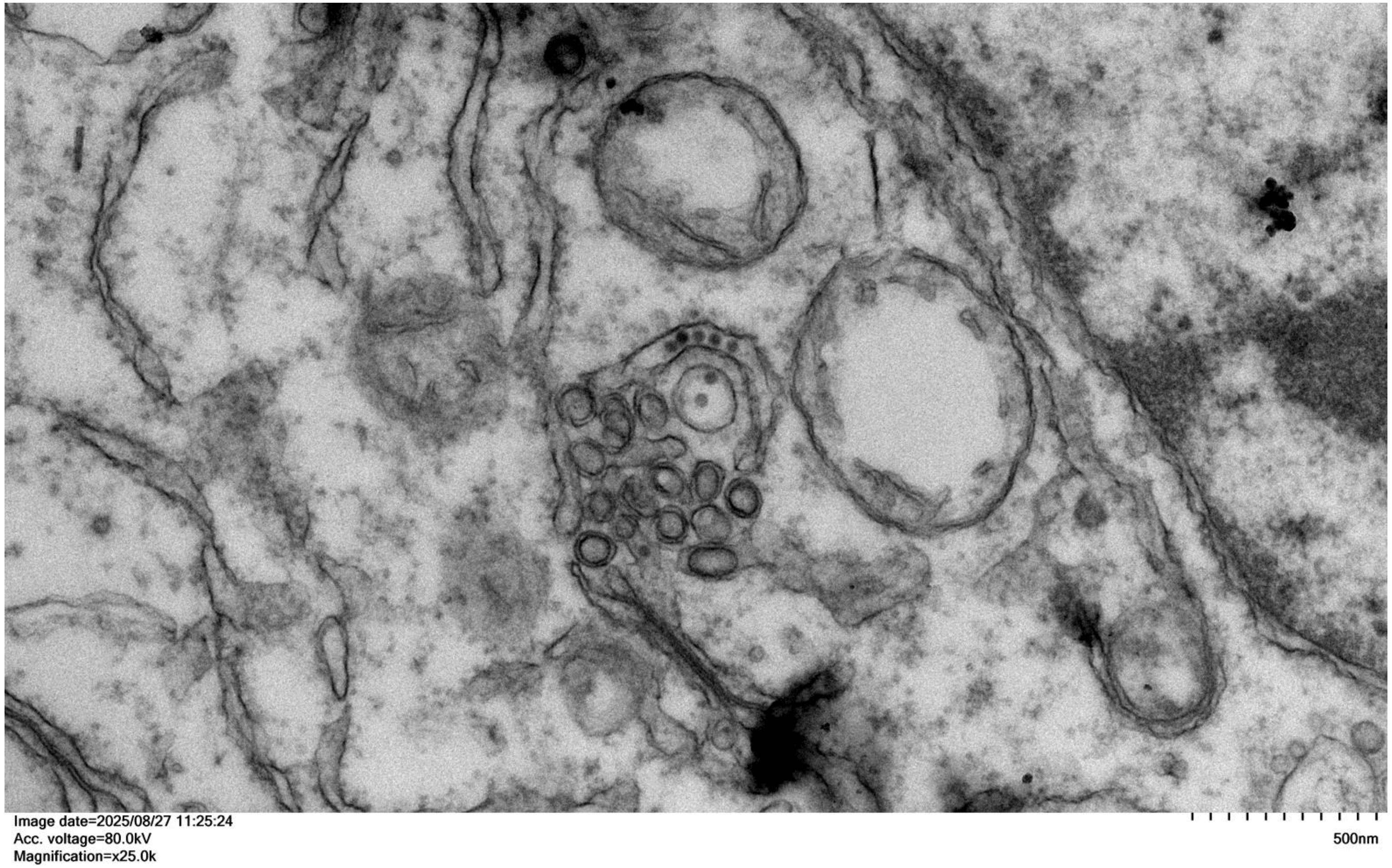

Figure 2F: Huh7 cells infected by dengue virus

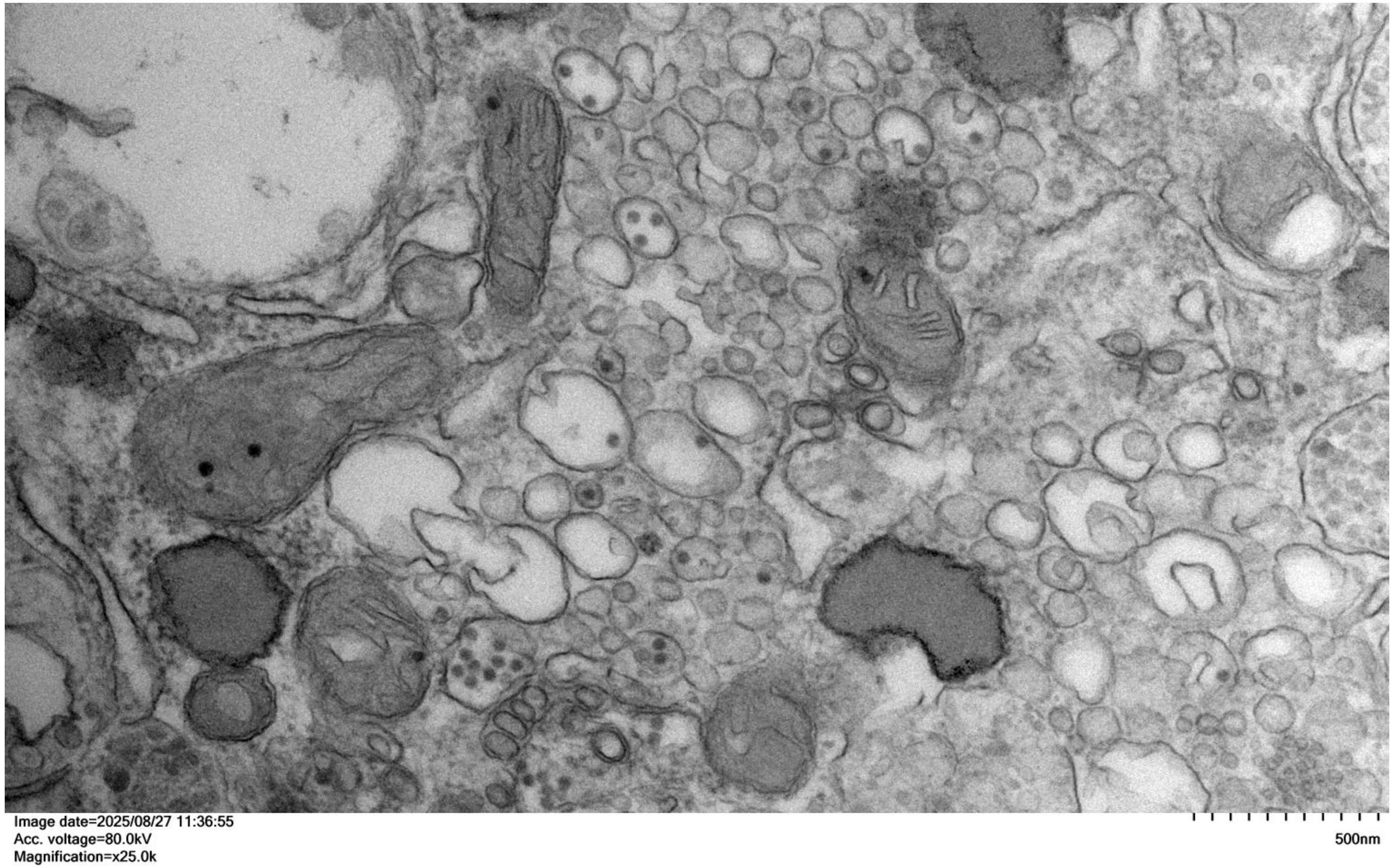

Representative TEM image of whole cell
Huh7 cells control non-infected

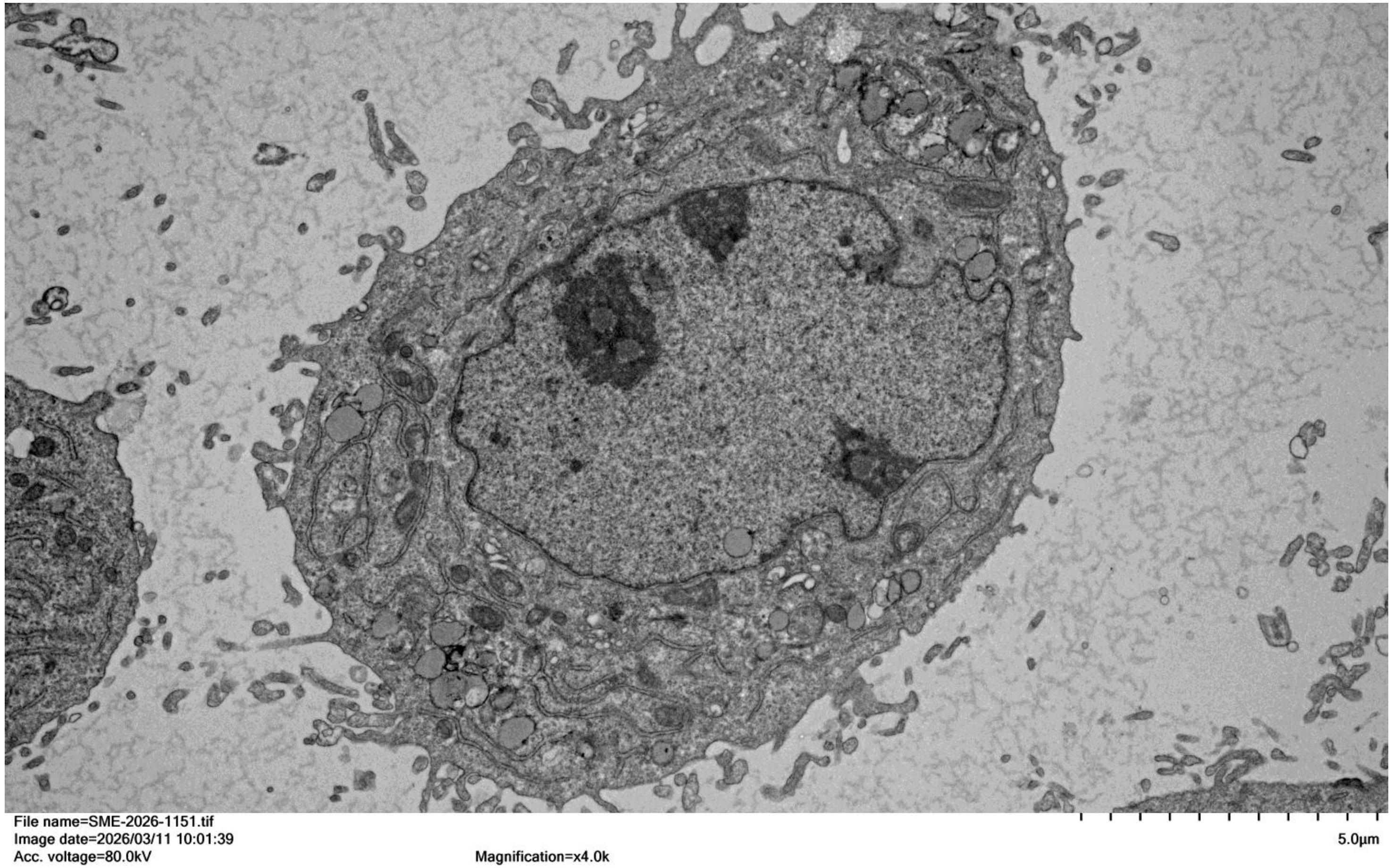

Figure 2A: Huh7 cells control non-infected

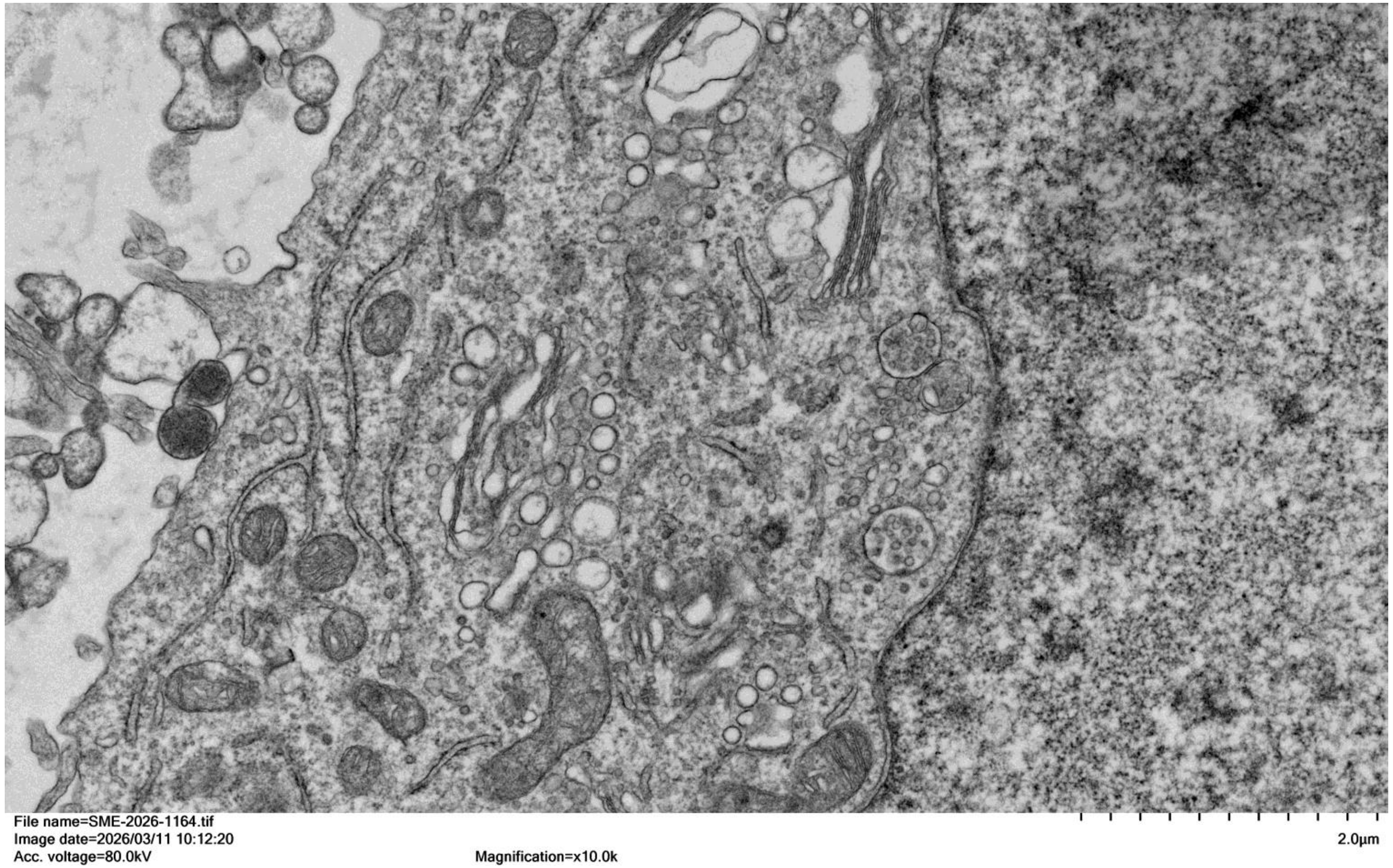

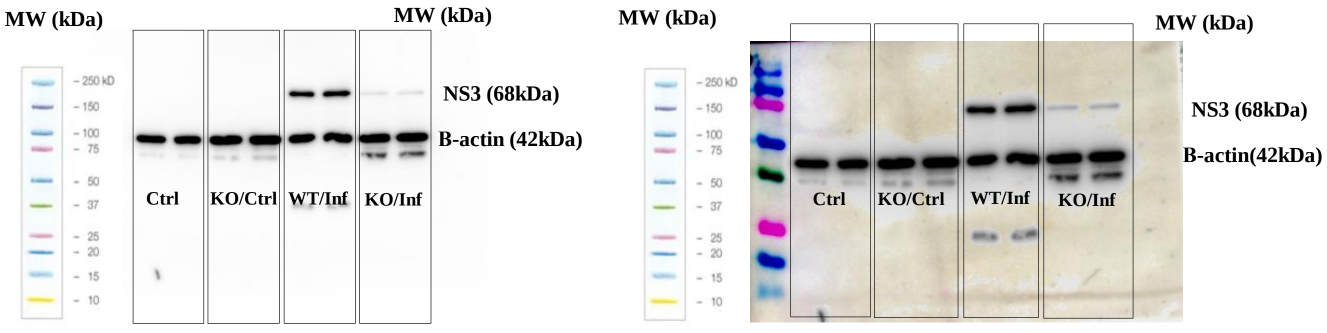

Figure S2. RTN3 knockout reduces dengue virus infection and RTN3S expression.

Left panel: Figure S2. Western blot membrane of NS3 (~68 kDa) with anti-NS3 (GTX124252; 1:1000; GenTex) and anti-β-actin (20536-1-AP; 1:20000; ProteinTech) antibodies, respectively. Gel-separated proteins were transferred to PVDF (0.45 µm pore size; Immobilon®-P Membrane) by wet/tank transfer systems electroblotting (100 V/350 mA, 60 min). Membranes, incubated with a horseradish peroxidase-conjugated secondary antibody (31460, 31430; 1:5000; ThermoFisher scientific), were developed with clarity western ECL substrate (Biorad). #Weight marker (molecular weight in kDa):Precision Plus Protein™ Kaleidoscope™ Prestained Protein Standards, BioRad,10 to 250 kDa; catalogue number: 1610375. Equal amounts of protein (50 µg per lane), quantified by BCA assay, were loaded on 10% . Sep 2025.

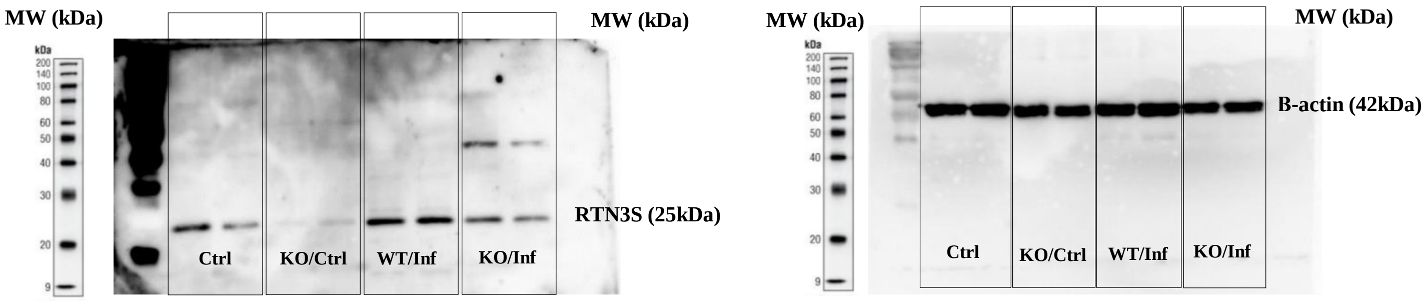

Figure S2. RTN3 knockout reduces dengue virus infection and RTN3S expression.

Left panel: Figure S2. Western blot membrane of RTN3 (~25 kDa) protein detected with anti-RTN3 (12055- 2-AP; 1:1000; ProteinTech) and anti-β-actin (20536-1-AP; 1:20000; ProteinTech) antibodies, respectively. Gel-separated proteins were transferred to PVDF (0.45 µm pore size; Immobilon®-P Membrane) by wet/tank transfer systems electroblotting (100 V/350 mA, 60 min). Membranes, incubated with a horseradish peroxidase-conjugated secondary antibody (31460, 31430; 1:5000; ThermoFisher scientific) and Anti-biotin HRP-linked Antibody (7727;1/1000; Cell signaling) , were developed with clarity western ECL substrate (Biorad). #Weight marker (molecular weight in kDa): Biotinylated protein ladder; , Cell signaling, 9 to 200 kDa; catalogue number: 7727. Equal amounts of protein (50 µg per lane), quantified by BCA assay, were loaded on 12% . Sep 2025.
